# Evaluating the impact of delayed study startup on accrual in cancer studies

**DOI:** 10.21203/rs.3.rs-3660904/v1

**Published:** 2024-04-19

**Authors:** Isuru Ratnayake, Anh-Tuan Do, Daniel Gajewski, Sam Pepper, Oluwatobiloba Ige, Natalie Streeter, Tara L. Lin, Matthew McGuirk, Byron Gajewski, Dinesh Pal Mudaranthakam

**Affiliations:** 1 Department of Biostatistics & Data Science, University of Kansas Medical Center, Kansas City, KS, USA.; 2 The University of Kansas Cancer Center– Accelerated Cancer Education (ACE) Summer Intern 2023, Piper High School, Kansas City, KS, USA.; 3 Department of Biostatistics & Data Science Summer Intern 2023, Rockhurst High School, Kansas City, MO, USA.; 4 The University of Kansas Cancer Center, Kansas City, KS, USA.

**Keywords:** clinical trials, study activation, trial accrual, study startup, oncology studies

## Abstract

**Background:**

Drug development in cancer medicine depends on high-quality clinical trials, but these require large investments of time to design, operationalize, and complete; for oncology drugs, this can take 8–10 years. Long timelines are expensive and delay innovative therapies from reaching patients. Delays often arise from study startup, a process that can take 6 months or more. We assessed how study-specific factors affected the study startup duration and the resulting overall success of the study.

**Method:**

Data from The University of Kansas Cancer Center (KUCC) were used to analyze studies initiated from 2018 to 2022. Accrual percentage was computed based on the number of enrolled participants and the desired enrollment goal. Accrual success was determined by comparing the percentage of enrollments to predetermined threshold values (50%, 70%, or 90%).

**Results:**

Studies that achieve or surpass the 70% activation threshold typically exhibit a median activation time of 140.5 days. In contrast, studies that fall short of the accrual goal tend to have a median activation time of 187 days, demonstrating the shorter median activation times associated with successful studies. Wilcoxon rank-sum test conducted for the study phase (W=13607, p-value=0.001) indicates that late-phase projects took longer to activate compared to early-stage projects. We also conducted the study with 50% and 90% accrual thresholds; our findings remained consistent.

**Conclusions:**

Longer activation times are linked to reduced project success, and early-phase studies tend to have higher success than late-phase studies. Therefore, by reducing impediments to the approval process, we can facilitate quicker approvals, increasing the success of studies regardless of phase.

## Introduction:

The study startup process is a crucial yet complex regulatory step in clinical trials that ensures quality and subject safety [1]. Common problems that can arise during study startup include a lack of clarity in contractual terms, financial negotiations, lack of funding availability, challenges in study design, delays in review committees, etc. Delays during the study startup phase are significant issues that can hinder trial success, as the time taken for study startup is critical for overall trial success [2]. In recent years, activation rates for cancer clinical trials in safety-net settings (those serving medically underserved populations) have significantly declined due to rising expenses and the inability to perform tasks such as study-related procedures, resulting in increased costs and trial complexity [3].

The study startup process requires the coordination of multi-disciplinary teams to ensure a comprehensive perspective throughout the process. A key element of this process is the local institutional scientific review, where study protocols are rigorously examined by Institutional Scientific Review Committees (SRCs) and Institutional Review Boards (IRBs) [1].

SRCs assess a study's design, scientific merit, alignment with the catchment area, and ethical considerations in advance of IRB review. They serve as an initial filter to advance only high-quality studies, but challenges such as limited staff, late submissions, and complex study design can lead to review delays during the startup process. Improving the effectiveness of SRCs requires communication, external incentives, senior-level support, and staffing enhancements. IRBs conduct a thorough review of study protocols, ensuring compliance with regulations, ethical standards, and institutional policies, while also ensuring the safety and privacy of study participants. The approval process through these review boards can be time-consuming: the National Cancer Institute (NCI) recommends a study startup time of 90 days from sponsor approval [4].

In the summer of 2020, The University of Kansas Cancer Center (KUCC) introduced a system known as Trial Review and Approval for Execution (TRAX). TRAX improves communication and documentation in order to increase the likelihood of meeting trial activation metrics. The KUCC’s regulatory process includes submission criteria, custom fields/pages, automated communication letters/notifications, review of meeting agendas and minutes, and dashboards for tracking key metrics. These features streamline the study startup process. Additionally, KUCC employs a study scoring mechanism that assigns scores based on scientific importance, portfolio, significance, funding, study type, and projected annual accrual.

Many novel drugs for treating cancer have undergone approval in recent years [5,6,7]. These were predicated upon the successful recruitment, completion, and design of multiple clinical trials [8]. However, clinical trial startup nevertheless proves to be quite complex, especially at academic medical centers.

## Methods:

The data were retrieved from the KUCC Study Startup Shiny R Dashboard, which displays and summarizes data from the Clinical Trial Data Management System (CTMS). Both systems are managed by the Department of Biostatistics & Data Science at The University of Kansas Medical Center.

The data consist of studies that were conducted by the KUCC including and between the January 1, 2018, and December 31, 2022. There were 720 new studies that entered the study startup process during this study period, and each study is categorized as either closed or enrolling. A ‘closed’ clinical trial has completed its accrual across all study sites; an ‘enrolling’ study has enrollment still in progress. Of the studies, 519 (72%) are closed studies, while the remaining 201 (28%) are ongoing studies.

The **Accrual Success** variable reflects the success of the accrual process (Equation 1). It is a dichotomous outcome variable (1=success; 0=fail), determined by measuring whether the percentage of enrolled patients meets a predefined threshold level (*k*) after study activation. A study is categorized as “successful” if it achieves or surpasses a predetermined *k* threshold; values of *k* ∈ {0.5,0.7,0.9} which are equivalent to 50%, 70%, and 90%respectively, were examined.


(1) $$Accrual\;Success=\left\{\begin{array}{c} 0:\frac{number\;of\;enrollments}{desired\;acrual\;goal}<k\\ 1:\frac{number\;of\;enrollments}{desired\;acrual\;goal}\geq k \end{array}\right..$$

It can be seen from Equation 1 that the percentage of enrollments determines Accrual Success. We excluded clinical trials from the analysis under several conditions: clinical trials that were still enrolling patients, and which had not yet met the threshold level (*k)* clinical trials with un-defined Accrual Success; clinical trials missing values of phase (i.e., Phase=N/A); and clinical trials where the number of days from Disease Working Group (DWG) to activation is unknown. After cleaning the data, there are 130 (42%), 116 (37%), and 79 (25%) studies labeled as successful based on a *k* of 0.5, 0.7, and 0.9, respectively.

The primary objective of this study is to determine if there exists an association between the time to study approval and the success in accruing participants for the study. This relationship is of significant interest, as it examines the impact of approval timelines on study outcomes and offers potential opportunities for improvement. To explore this association, we have introduced the variable **Activation Days**, which represents the business day interval between DWG approval and the study’s activation date. Any days that were on sponsor hold during this period have been deducted from the total Activation Days. This variable can be expressed as follows:

(2) Activation Days=A−B

Here,

$$A=f\left(studt\;activation\;date\;-\;DWG\;approval\;date\right),$$

$$B=f\left(sponsor\;hold\;end\;date\;-\;sponsor\;hold\;start\;date\right).$$

The function $f\left(.\right)$ in Equation 2 calculates the business days between any given calendar dates and returns a positive integer as the output; thus, this function excludes weekends and US holidays. For example, if a clinical trial received its DWG approval on October 7, 2022, and was activated on May 12, 2023, with 8 days on sponsor hold, Activation Days are equal to 152 business days. This calculation is explained as follows:

$$Activation\;Days=f\left(\text{May}\;12,2023-\text{October}\;7,2022\right)-f\left(\text{July}\;5,2022-\text{June}\;23,2022\right)=152$$

Iťs important to note that not all studies have experienced sponsor holds.

Another important objective of the study is to examine the association between the study phase (i.e., pilot, I, II, III, and IV) and the success of the accrual process. To investigate this, a dichotomized explanatory variable (early-phase, late-phase) is introduced and named **Study Phase**, which is described in Equation 3:

(3) $$Study\;Phase\;=\left\{\begin{array}{c} 0=early-phase:Phase\;\in \left(pilot,I,I/II\right)\\ 0=late-phase:Phase\;\in \left(II,II/III,III/IV\right) \end{array}\right..$$

These studies were submitted by different sources: to denote these various funding categories, the **Study Source** variable was introduced. This is a nominal categorical variable with 5 categories: Externally Peer-Reviewed/Federal Funding, Industrial/Pharmaceutical, Internal Institutional/Investigator Initiated, External Institutional/Investigator Initiated, and National/Cooperative Group/Consortium.

We conducted a comprehensive statistical analysis using various techniques. Initially, we constructed box-and-whisker plots for the Accrual Success and Study Phase variables to visually assess the distribution of Activation Days. Given the non-normal distribution of the data, we applied the Wilcoxon rank-sum test to compare the median Activation Days between the Accrual Success and Study Phase groups, and we used the Kruskal-Wallis test to compare the median Activation Days among different Study Source groups. Furthermore, we employed Pearson’s chi-squared test to investigate potential pairwise associations between Accrual Success and Study Phase, and Fisher's exact test to assess potential association between Accrual Success and Study Source due to the lower number of observations (< 5) in some categories. To study factors affecting Accrual Success, we fitted generalized linear models, considering variables such as the Activation Days and the Study Phase. Model selection was based on the Akaike Information Criteria (AIC). We also computed 95% confidence intervals and odds ratios for the chosen model. All statistical analyses were conducted using specific packages in R (version 4.3.1) including dplyr [9], ggpubr [10], gridExtra [11], janitor [12], and RColorBrewer [13].

## Results:

The minimum and maximum values for Activation Days recorded during the study period are 13 and 681 business days, respectively. The median number of Activation Days is 172.5, while the mean is 194.8, both of which are shorter than a business year consisting of 252 days. The distribution of Activation Days is positively skewed (1.13), and the Shapiro-Wilk test for normality (S=0.92, p-value<0.001) indicates that the distribution for Activation Days is not normally distributed.

**Figure 1:**
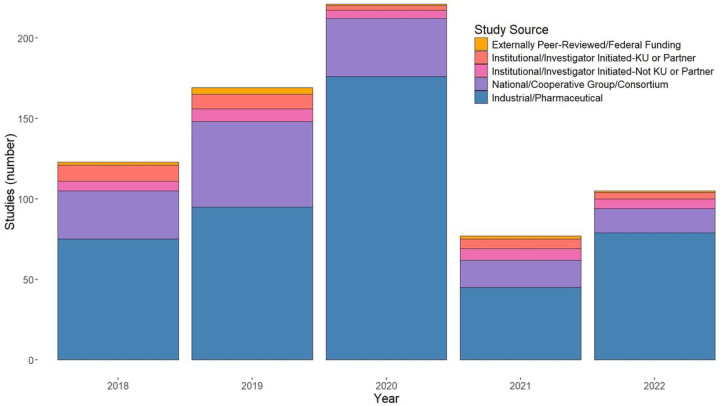
Trends in Studies by Study Sources (2018–2022)

According to Figure 1, the number of studies received by the KUCC increased steadily from 2018 to 2020. However, there was a 65% sudden drop in 2021, followed by 27% increase in 2022; this may be due to the persistent effects of the COVID-19 pandemic. Most of the studies (>50%) were received from Industrial/Pharmaceutical sponsors, with the second-highest study source being National/Cooperative Group/Consortium; Externally Peer-Reviewed/Federal Funding made up the smallest portion of studies for each year. Additionally, each study is categorized as either early-phase or late-phase, as described in Equation 3. Most projects received over this period were late-phase studies.

**Figure 2:**
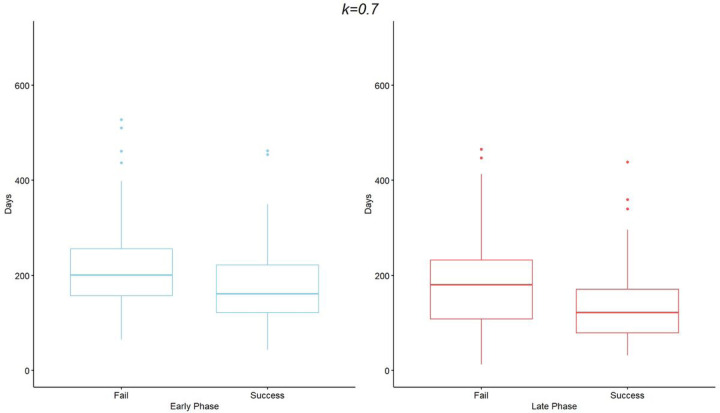
Box and Whisker Plots of Activation Days for Accrual Success by Study Phase

In this study we utilized three different threshold values (*k* = {0.5,0.7,0.9}) to define the Accrual Success variable. The remainder of these results are based on the *k* = 0.7 threshold value; the results for threshold values of *k* = 0.5 and *k* = 0.9 may be found in the Supplementary Materials. Once a study successfully reaches its accrual goal, it typically takes a median of 140.5 Activation Days. Conversely, studies that fail to meet their accrual goal experience a longer median of 187 Activation Days. Based on the Wilcoxon rank-sum test results (W=14058, p-value <0.001), it can be concluded at level 0.05 significance level that the median Activation Days tend to be higher for the failed studies than the successful studies. Furthermore, the results of the Wilcoxon rank-sum test conducted for the study phase (W=13607, p-value=0.001) indicate, at level 0.05, that the median Activation Days for late-phase projects are greater than those for early-phase projects. The Kruskal-Wallis test statistic indicates a significant difference in median Activation Days among the various sources of study data (H=93.21, p value <0.001). Figure 2 illustrates that, across both early-phase and late-phase studies, successful studies consistently have lower median Activation Days compared to their unsuccessful counterparts.

Table 1 present the Pearson’s chi-squared test results for the pairwise association between Accrual Success and Study Phase, and Table 2 shows the Fisher’s exact test results for the association between Accrual Success and Study Source. According to Pearson’s chi-squared test, it can be concluded with 95% confidence that there is a significant association between Study Success and Study Phase. However, Fisher’s exact test suggests that there is no evidence to support a significant association between Study Source and Accrual Success with 95% confidence.

We fitted a logistic regression model to explore the linear relationship between the logit of the probability of Accrual Success (*Y*) and predictor variables, including Study Phase (*X*_1_), Activation Days (*X*_2_), and Study Source (*X*_3_). Based on the AIC model selection criteria, the following model was selected. More results can be found in Table 3.


(4) $$\text{logit}\left(P\left(Y=Yes|X_{1},|X_{2}\right)\right)=0.842-0.902*X_{1}-0.005*X_{2}.$$

According to the results in Table 3 it can be concluded, with 95% confidence, that as the number of Activation Days increases, the odds of success for a study decrease by 0.5% per day. Additionally, the odds of success for late-phase studies are approximately 41% of those for early-phase projects. In other words, as the time to activate a study increases, the odds of project success decreases, and early-phase projects exhibit higher odds of success compared to late-stage projects.

**Figure 3:**
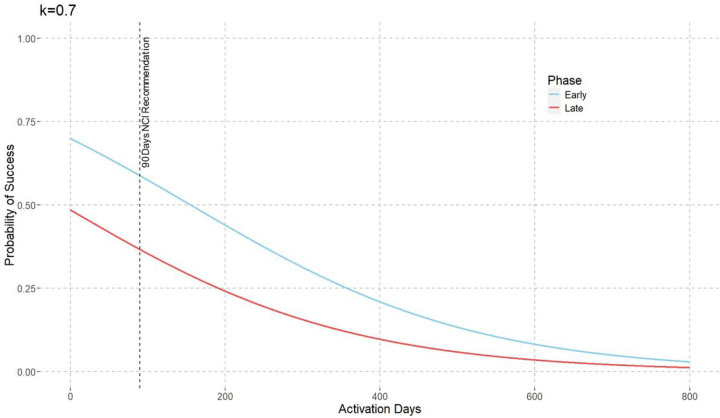
Probability of Success vs. Activation Days by Study Phase

Figure 3 illustrates the probability of success and how it varies with Activation Days and Study Phase. The probability of success decreases as the Activation Days increase, and early-phase projects exhibit a higher probability of success compared to late-phase projects.

Based on NCI recommendations, the ideal study startup time is 90 days. Using the model equation (Equation 4), we can calculate that the probability of success for early-phase projects is higher than that for late-phase projects.


$$P\left(Y=Yes|X_{1}=late-phase,\;X_{2}=90\right)=0.38,$$

$$\begin{array}{l} P\left(Y=Yes|X_{1}=early-phase,\;X_{2}=90\right)=0.60. \end{array}$$

## Discussion:

A team effort is required from Principal Investigator, study sponsor, regulatory teams, and review board to minimize this study start up times. Studies that have clearly define the goals of their research study, inclusion exclusion criteria, scope, recruitment strategies, and consent documents often obtain IRB approvals in the stipulated time period. Strategies to improve protocol obedience and participants' retention may include enrolling people at early disease stages [14]. A local institutional scientific review process should be necessary as part of the careful review of study protocols by IRBs [1]. The DWG members review these protocol documents and choose the study’s outcome: Approved, Rejected, or Tabled. Tabled studies may need further justification in their proposal, or they might be postponed or cancelled due to other ongoing studies competing with them. Alongside committee review centralized coverage analyses for multisite clinical trials have been shown to reduce risk for sites and patients, predict budget requirements, and help shorten trial startup times that hinder patient accrual and access to trials [15]. At The University of Kansas Cancer Centre (KUCC) we constructed a study scoring mechanism to allocate studies based on: 1) scientific importance (e.g., anywhere between phases I and III); 2) portfolio (completes “gap” studies); 3) significance (e.g., contribution to science); 4) funding (external, internal, or no funding); 5) study type (e.g., consortium or federal); and 6) projected annual accrual. Priority is categorized into: >50 (high); 30–50 (medium); and <30 (low). Studies that have a higher score get prioritization during the startup process minimizing delays by focusing the Clinical Trials office on what is deemed important. Continuing to innovative methods like these helps to minimize delays [3]. Single-center studies have been shown to have shorter activation time than multi-center studies [16]. The COVID-19 pandemic interrupted new trial startups, patient recruitment, and follow up visits for trials, resulting in major interruptions in cancer center trial unit operations [17]. Travel bans, quarantines, and stay-at-home orders naturally lead to fewer patient visits. New cancer clinical trial practices adopted during the COVID-19 pandemic include risk assessment strategies, remote trial coordination, data collection, and cheaper methods for future clinical trial conduct [18]. Randomized controlled rials are known as the “gold-standard” to assess the efficacy and safety of possible therapies or medications [19].

## Conclusion:

Study startup is a crucial step for every clinical trial, and developing a robust process to streamline regulatory, contracting, and financial negotiations is essential for achieving quicker study activation. The initial study startup time can often disrupt the momentum of the study team, resulting in delays. To address this, Cancer Centers nationwide should establish process repositories that set clear expectations for sponsors and investigators regarding timelines. Implementing a study tracker dashboard, similar to KUCC, can enhance transparency and help identify and address bottlenecks. It may also be necessary for Cancer Center leadership to make strategic decisions about pursuing studies based on their potential for quick activation. Furthermore, introducing scoring metrics for study intake to prioritize study activation can help manage workload and staffing challenges. By addressing barriers that impede the approval process, we can facilitate quicker approvals, ultimately increasing the success of studies regardless of their phase.

## Supplementary Material

Supplement 1

## Figures and Tables

**Table 1:** Chi-Square Test Results for Association Between Accrual Success and Study Phase.

Study Phase	Accrual Success	
	fail	success
early-phase	62 (56%)	48 (44%)
late-phase	147 (72%)	58 (28%)
*Pearson’s χ*^*2*^ *test p – value* = 0.0087		

The chi-squared test indicates significant association between Accrual Success and Study Phase.

**Table 2:** The Fisher’s Test Results for Association Between Accrual Success and Study Source.

Study Source	Accrual Success	
	fail	success
Externally Peer-Reviewed/Federal Funding	2 (50%)	2 (50%)
Industrial/Pharmaceutical	137 (69%)	62 (31%)
Institutional/Investigator Initiated: Internal	7 (54%)	6 (46%)
Institutional/Investigator Initiated: External	12 (86%)	2 (14%)
National/Cooperative Group/Consortium	51 (60%)	34 (40%)
*Fisher’s Exact test p – value* = 0.1801		

The Fisher's test shows no significant association between Accrual Success and Study Source.

**Table 3:** Logistic Regression Model Results for Accrual Success.

Covariate		N	Odds Ratio	95% Confidence Interval	p-value
	early	110	*Reference category*		
Study Phase	late	205	0.406	(0.242, 0.676)	<0.001
Activation Days		315	0.995	(0.991, 0.997)	<0.001

Early-phase was used as the reference group, both Study Phase and Activation Days are significant terms.

## List of abbreviations:

KUCC: The University of Kansas Cancer Center
SRCs: Scientific Review Committees
IRBs: Institutional Review Boards
NCI: National Cancer Institute
TRAX: Trial Review and Approval for Execution
CTMS: Clinical Trial Data Management System
DWG: Disease Working Group
AIC: Akaike Information Criteria
