# Supplementary material for: Evaluating the impact of delayed study startup on accrual in cancer studies
Source: Res Sq. 2024 Apr 19:rs.3.rs-3660904. Preprint. [Version 1] doi: 10.21203/rs.3.rs-3660904/v1 (PMC11065059; doi:10.21203/rs.3.rs-3660904/v1)
Supplement: Supplement 1 [file NIHPPRS3660904v1-supplement-1.pdf]

Supplementary Files

This is a list of supplementary files associated with this preprint. Click to download.

- AppendixFigureS1.jpg
- AppendixFigureS2.jpg
- AppendixFigureS3.jpg
- AppendixFigureS4.jpg
- SupplementaryMaterial.docx
